# Soft Computational Approaches for Prediction and Estimation of Software Development

**DOI:** 10.1155/2016/3905931

**Published:** 2016-02-18

**Authors:** Xiao-Zhi Gao, Arun Kumar Sangaiah, Muthu Ramachandran

**Affiliations:** ^1^Department of Electrical Engineering and Automation, Aalto University, 00076 Espoo, Finland; ^2^School of Computing Science and Engineering, VIT University, Vellore 632014, India; ^3^School of Computing and Creative Technologies, Leeds Beckett University, Leeds LS1 3HE, UK

During the past decades, the use of soft computational approaches has been extended to a large variety of software engineering applications. Soft computational methods, such as fuzzy systems, neural networks, evolutionary computation, and probability models including Bayesian network and chaos theory, are currently attractive topics in the extensive software engineering research problems. This special issue provides a platform for the dissemination of knowledge on both the empirical research and applied research in the field of soft computing. The objective of the special issue is to facilitate a forum to a wide spectrum of articles that cover the state-of-the-art techniques and recent results in the research of soft computational approaches. In particular, the special issue focuses on publishing the highly technical articles describing the software development topics: advanced software engineering, computational intelligence, and wireless sensor networks. This special issue has received overwhelming responses from researchers, and it has received a total of 28 high-quality submissions from various countries around the world. All the submitted papers have been evaluated by at least three independent reviewers. However, due to focused scope of the special issue, only a few manuscripts are published. Inevitably, hard editorial decisions had to be made, and some high-quality articles could not even be included. We believe that this special issue presents cohesive information related to the applications of soft computing methods in software development, and it also provides stimulations for future research.

In the paper entitled “An Enhanced PSO-Based Clustering Energy Optimization Algorithm for Wireless Sensor Network,” C. Vimalarani et al. propose a novel Enhanced PSO-Based Clustering Energy Optimization (EPSO-CEO) algorithm for the wireless sensor network (WSN), in which the clustering and clustering head selection are done by using the Particle Swarm Optimization (PSO) algorithm with respect to minimizing the power consumption in the WSN.

The results obtained have been compared and evaluated on the basis of energy efficiency.

The paper of Y. Rastegari and F. Shams entitled “Optimal Decomposition of Service Level Objectives into Policy Assertions” presents a method to decompose service level objectives to web service policy assertions. Transformation of Web Service Choreography Description Language (WS-CDL) to Web Service Business Process Execution Language (WSBPEL) has been addressed in some related works, but neither of them considers quality aspects of transformation or run-time adaptation. In this paper, in conformity with web services standards, the authors proposed an optimal decomposition method to make a set of WS-policy assertions. Assertions applied to WSBPEL elements and affect their run-time behaviors. The decomposition method achieves the best outcome for a performance indicator. It also guarantees the lowest adaptation overhead by reducing the number of service reselections. This research considered securities settlement case study to prototype and evaluated the decomposition method. The results show an acceptable threshold between customer satisfaction, the targeted performance indicator through the case study, and adaptation overhead.

G. J. Eanoch and T. S. Sankar present a Neuro-Fuzzy Energy Aware Clustering Scheme (NFEACS) in their paper entitled “Development of Energy Efficient Clustering Protocol in Wireless Sensor Network Using Neuro-Fuzzy Approach” to form the optimum energy aware clusters. The proposed method consists of two part, fuzzy subsystem and neural network system, which can yield energy efficiency in building up clusters and cluster heads in the WSN. More precisely, it uses neural network that provides effective training sets related to energy and received signal strength of all nodes in order to estimate the expected energy for tentative cluster heads. The sensor nodes with higher energy are trained with the center location of base station for selecting the energy aware cluster heads. Fuzzy IF-THEN mapping rules are also used so as to find the clusters and cluster heads. Experiment results show that the NFEACS performs better than the existing solutions.

In the paper entitled “Software Design Challenges in Time Series Prediction Systems Using Parallel Implementation of Artificial Neural Networks,” the authors (N. Manikandan and S. Subha) develop a comprehensive approach to architectural design for financial prediction models. During the past two decades, a large number of artificial neural networks-based learning models have been studied to manipulate with financial data, for example, prediction of the future trends and prices. This paper focuses on the architectural design related issues for performance improvement through vectorising the strengths of multivariate econometric time series models and artificial neural networks. It further provides an adaptive and hybrid method for predicting the exchange rates.

In the paper entitled “Energy-Aware Multipath Routing Scheme Based on Particle Swarm Optimization in Mobile Ad Hoc Networks” written by Y. H. Robinson and M. Rajaram, a novel energy aware multipath routing scheme is proposed, which is based on the Particle Swarm Optimization (EMPSO) that uses Continuous Time Recurrent Neural Network (CTRNN) to deal with optimization problems. The CTRNN has been applied to find the optimal loop-free paths for solving the link disjoint paths in MANET. In other words, it is used as an optimum path selection technique to produce a set of optimal paths between source and destination. In the CTRNN, the PSO method is particularly utilized for training the RNN. The proposed scheme uses the reliability measures, for example, transmission cost, energy factor, and the optimal traffic ratio between source and destination, to increase the routing performance. Therefore, the optimal loop-free paths can be discovered using the PSO to seek better link quality nodes in the route discovery phase suitable for the dynamic topology changing property of a MANET.

